# Machine learning integrates metabolomics and proteomics to identify key regulators of anthocyanin biosynthesis in edible rose petals

**DOI:** 10.3389/fpls.2026.1751780

**Published:** 2026-03-13

**Authors:** Dannuo Fu, Jian Chen, Shuang Liang, Hongwei Fu

**Affiliations:** 1 College of Life Sciences and Medicine, Zhejiang Sci-Tech University, Hangzhou, China; 2 Hangzhou Judu Technology Co., Ltd, Hangzhou, China; 3 Beijing University of Chinese Medicine, Beijing, China

**Keywords:** anthocyanin, edible roses, functional food, metabolomics, proteomics, transcription factor

## Abstract

Edible rose petals represent a promising source of anthocyanins, natural pigments with health-promoting properties suitable for functional food applications. However, the molecular mechanisms regulating anthocyanin biosynthesis in roses remain incompletely understood. Here, we employed an integrated metabolomic and proteomic approach to investigate anthocyanin profiles and their regulatory networks in three edible rose varieties: Rosa alba (RA), Rosa damascena (RD), and Rosa centifolia (RC). We identified a total of 13 anthocyanins, with RC exhibiting the highest total anthocyanin content—6.9% higher than RD and fivefold greater than RA. Compositional analysis revealed variety-specific accumulation patterns: RD was rich in delphinidin and peonidin derivatives, while RA predominantly accumulated pelargonidin-3-O-rutinoside. Proteomic analysis identified 9,924 proteins, and weighted gene co-expression network analysis (WGCNA) highlighted the MEblue module as strongly correlated with anthocyanin accumulation. By integrating a KNN-based machine learning model, we identified ten key structural proteins (e.g., RhUFGT, F3H, DFR) and several transcription factors (e.g., *NAC*, *bZIP_2*, *C3H*) as central regulators. Our findings elucidate the molecular basis of anthocyanin biosynthesis in edible roses and provide potential targets for breeding strategies aimed at enhancing their value as functional food ingredients.

## 1 Introduction

Growing public health awareness has positioned functional foods as a prominent consumer trend. Among these, functional beverages are particularly popular for their pleasant taste and convenience. Anthocyanins, a class of natural compounds with validated potent antioxidant activity, have been demonstrated to offer multiple health benefits, including anti-inflammatory and cardiovascular-protective effects (Zhang et al., 2014; Martín et al., 2017), thereby establishing them as core functional ingredients in beverages. Currently, anthocyanin-rich drinks are especially favored by adolescent consumers for their recognized roles in antioxidation, vision protection, and overall wellness.

Anthocyanins, the principal water-soluble pigments in the flavonoid family, are widely present in the petals and fruits of angiosperms—including cherries, orchids, roses, and rhododendrons. To date, more than 650 distinct anthocyanins have been identified, predominantly falling into six major classes: delphinidin, cyanidin, pelargonidin, peonidin, petunidin, and malvidin. These compounds not only create vibrant red, purple, and blue hues in plant tissues but also fulfill essential physiological functions such as facilitating seed dispersal, providing UV protection, and enhancing resistance to environmental stresses like cold and drought. Moreover, extensive research has confirmed that anthocyanins exhibit a broad spectrum of biological activities—including anti-inflammatory (Burgos-Edwards et al., 2019; Kim et al., 2009; Li et al., 2019), antioxidant (Zhang et al., 2020; Li et al., 2020), anticancer (Lala et al., 2006; de Ferrars et al., 2014; Kalt et al., 2020), immunomodulatory (Min et al., 2015; Ramirez-Tortosa et al., 2001), antibacterial (Cisowska et al., 2011), and anti-aging (Wang et al., 2024) effects, as well as cardioprotective (Reis et al., 2016) and metabolic regulatory properties related to blood glucose and lipids (Herrera-Balandrano et al., 2021), highlighting their considerable potential in health promotion and disease prevention.

Edible flowers, serving as a significant source of anthocyanins in functional beverages, have been integral to culinary traditions across various cultures since antiquity, including ancient Rome, Greece, and China (Takahashi et al., 2020). The advancement of analytical techniques, particularly Ultra-High Performance Liquid Chromatography-Tandem Mass Spectrometry (UHPLC-MS/MS), has greatly facilitated the identification of chemical constituents and biological properties of edible flowers. Consequently, they are no longer merely plate decorations but are increasingly recognized as innovative natural resources for the development of bioactive ingredients (Hegde et al., 2022). This evolution provides new perspectives for designing functional foods rich in anthocyanins.

Globally distributed throughout temperate and subtropical regions of the Northern Hemisphere, roses (*Rosa* spp.) are key ornamental plants with a prominent role in horticulture, perfumery, and functional product development, owing to their vibrant colors, elegant morphology, and distinctive fragrance. Beyond their visual and olfactory appeal, edible roses are also valued in both traditional medicine and modern functional foods. Their petals contain a range of bioactive compounds—such as anthocyanins, flavonoids, polyphenols, and vitamin C—which contribute to antioxidant, anti-inflammatory, and soothing properties.

In recent years, advances in metabolomics and molecular biology have made the biosynthesis pathways of secondary metabolites—such as anthocyanins and terpenes—and their regulatory mechanisms in roses a major research focus. These studies provide a scientific foundation for the high-value utilization of roses in natural pigments, health foods, and cosmetics. Notably, roses are rich in bioactive compounds such as flavonoids, polysaccharides, vitamin C, and essential amino acids. Combined with their distinctive aromatic properties, these components confer notable health benefits and edible value to rose-based products. It is precisely for these reasons that foods developed from rose petals and fruits—including pastries, jams, and salads—are gaining increasing popularity in the international market (Hegde et al., 2022; Zhou et al., 2024).

Regarding the color mechanism, anthocyanins serve as the principal pigments responsible for the coloration of flowers and fruits. In general, cyanidin and pelargonidin impart red hues, peonidin contributes to purplish-red tones in plant tissues, while delphinidin, petunidin, and malvidin are associated with blue and bluish-purple shades (Khoo et al., 2017). In roses, anthocyanins—a class of water-soluble flavonoids present in petals—confer vibrant red, purple, pink, and other colors. The major anthocyanidins identified in roses include cyanidin, delphinidin, pelargonidin, and peonidin. Notably, methylated derivatives of these anthocyanins, such as methylated peonidin and delphinidin, not only improve pigment stability and shift color expression toward blue-purple and magenta but also often exhibit enhanced biological activity. As a result, anthocyanin content and composition have become key criteria for evaluating rose quality. Different varieties possess distinct “anthocyanin fingerprints,” and those with bright, stable coloration tend to be more competitive in the market. For edible roses, determining the optimal harvest timing is closely linked to the peak period of anthocyanin accumulation.

At the molecular level, anthocyanin biosynthesis is finely regulated by several families of transcription factors (TFs). Studies have shown that TFs such as *MYB*, *bHLH*, *WD40*, *bZIP*, and *MADS-box* influence anthocyanin synthesis by binding to the promoter regions of structural genes (An et al., 2017; Jian et al., 2019; Khan et al., 2022; Lu et al., 2018). Among these, the regulatory roles of the *MYB*, *bHLH*, and *WD40* families are particularly well characterized. These TFs can function independently or assemble into the MBW (*MYB–bHLH–WD*) protein complex to exert their effects (Feng et al., 2020; Xu et al., 2015). MYB transcription factors are generally regarded as the most pivotal regulators of anthocyanin biosynthesis in plants. A large number of *R2R3-MYB* TFs have been identified, most of which act as positive regulators, though a minority function as repressors (Butelli et al., 2012; Pratyusha and Sarada, 2022; Ni et al., 2020). However, current research on the regulatory mechanisms of anthocyanin synthesis in roses remains largely concentrated on the *MYB* family, while the roles of other types of transcription factors are still poorly understood. This knowledge gap limits a comprehensive and in-depth interpretation of the molecular basis of rose coloration.

In this study, based on the significant differences in petal color and commercial availability, we selected three edible rose varieties - Rosa alba (RA), Rosa damascena (RD), and Rosa centifolia (RC). These common edible rose varieties represent different ranges of anthocyanin accumulation, providing an ideal system for studying the molecular basis of color formation and pigment diversity. By integrating metabolomic (UPLC-MS/MS) and proteomic approaches, we systematically analyzed the variations in anthocyanin composition and content among these varieties, aiming to elucidate the molecular mechanisms responsible for their differential anthocyanin accumulation. Through correlation analysis of multi-omics data, key proteins and transcription factors involved in anthocyanin biosynthesis were identified. This research not only expands the regulatory network of anthocyanin biosynthesis in roses but also provides potential genetic targets for breeding programs. Our work offers a scientific foundation for the industrial utilization of rose petals as sustainable sources of natural pigments and functional food ingredients.

## 2 Materials and methods

### 2.1 Plant materials

Rose plants were cultivated in the experimental field located in Sanyuan County, Xianyang City, Shaanxi Province, China (34°41′05″ N, 108°56′15″ E). Flower bud sampling was conducted between 9:00 and 10:00 AM in May 2023. To account for biological variation, petals were collected from 50 individual flower buds per variety, which were randomly selected from different plants across the cultivation area. These 50 petal samples were pooled to form one biological replicate. A total of three independent biological replicates (each from a separate batch of 50 buds harvested on different days) were prepared for each variety for metabolomic analysis. The remaining petal materials were immediately frozen in liquid nitrogen and stored at -80 °C for subsequent proteomic sequencing.

### 2.2 Widely targeted metabolomics analysis

Frozen rose samples were placed in a freeze-dryer (Scientz-100F) for vacuum freeze-drying, then ground into powder using a grinding machine (MM 400, Retsch) at 30 Hz for 1.5 minutes. 50 mg of sample powder was weighed using an electronic balance (MS105DM), and 1200 μL of -20 °C pre-cooled 70% methanol aqueous internal standard extraction solution was added. The mixture was then vortexed every 30 minutes for 30 seconds each time, totaling 6 times. Subsequently, it was centrifuged at 12000 rpm for 3 minutes, the supernatant was aspirated, and the sample was filtered through a 0.22 μm microporous filter membrane. Equal volumes of each sample were combined to prepare a pooled quality control (QC) sample, which was stored in vials for UPLC-MS/MS analysis. Data acquisition used an ExionLC™ AD UPLC system and a tandem mass spectrometry system, with an Agilent SB-C18 column (1.8 μm, 2.1 mm * 100 mm). For liquid chromatography conditions, mobile phase A was ultrapure water with 0.1% formic acid, and B was acetonitrile with 0.1% formic acid. The gradient conditions were as follows: 0.00 - 9.00 min, 5 - 95% B; 9.00 - 10.00 min, 95% B; 10.00 - 11.10 min, 95 - 5% B; 11.10 - 14.00 min, maintain 5% B for equilibration. The column temperature was 40 °C, flow rate 0.35 mL/min, and injection volume 2 μL. Mass spectrometry conditions were: ESI temperature 500 °C, ion spray voltage 5500 V for positive ion mode and -4500 V for negative ion mode, ion source gas I, II, and curtain gas set to 50, 60, and 25 psi, respectively. QQQ scanning used MRM mode, with specific MRM ion pairs monitored based on optimized declustering voltage and collision energy for different eluting metabolites.

### 2.3 Proteomics analysis

Two grams of frozen sample were pulverized in liquid nitrogen, transferred to a centrifuge tube, and combined with L3 lysis buffer—containing 1% SDS, 100 mM Tris-HCl, 7 M urea, 2 M thiourea, 1 mM PMSF, and 2 mM EDTA—at a 1:5 (w/v) ratio. After thorough vortexing, the mixture was ultrasonically lysed on ice for 10 min, followed by centrifugation to collect the supernatant as the total protein extract.

The protein solution was then treated with four volumes of prechilled acetone and precipitated overnight at –20 °C. After centrifugation at 4 °C, the pellet was retained, washed with cold acetone, and redissolved in 8 M urea. Total protein concentration was determined using the BCA assay. An aliquot of protein solution was adjusted to 200 μL with 8 M urea, reduced with 10 mM DTT at 37 °C for 45 min, and alkylated with 50 mM iodoacetamide (IAM) for 15 min at room temperature in the dark. The proteins were then reprecipitated with four volumes of prechilled acetone at –20 °C for 2 h, centrifuged, and air-dried.The resulting pellet was resuspended in 200 μL of 25 mM ammonium bicarbonate containing 3 μL trypsin (Promega) and digested overnight at 37 C. The resulting peptides were desalted using C18 solid-phase extraction, concentrated by vacuum centrifugation, and reconstituted in 0.1% (v/v) formic acid for LC-MS/MS analysis.Chromatographic separation was performed on a NanoElute ultra-high-performance liquid chromatography system, and mass spectrometric data were acquired using a timsTOF Pro2 instrument.

### 2.4 Bioinformatical analysis

In the proteomic analysis, each experimental group included three biological replicates. Protein identification required the detection of at least one unique peptide, with a false discovery rate (FDR) of ≤ 0.01 applied for peptide validation. Differentially abundant proteins were defined as those exhibiting a fold change >1.5 or <0.67 with a P-value < 0.05. Transcription factors (TFs) were predicted using iTAK software, and protein interaction networks were visualized with Cytoscape (version 3.7.2).

All statistical analyses were performed in R. K-means clustering was conducted using R 4.2.0 with the stats package (v4.2.0), while Weighted Gene Co-expression Network Analysis (WGCNA) was implemented with R 4.2.2 and the WGCNA package (v1.71). Machine learning analyses utilized R 4.5.1. Correlations between proteins and transcription factors were assessed using Pearson correlation coefficients (PCC), with interactions having PCC > 0.9 considered significant.

### 2.5 Quantitative real-time polymerase chain reaction analysis

**Figure 1:**
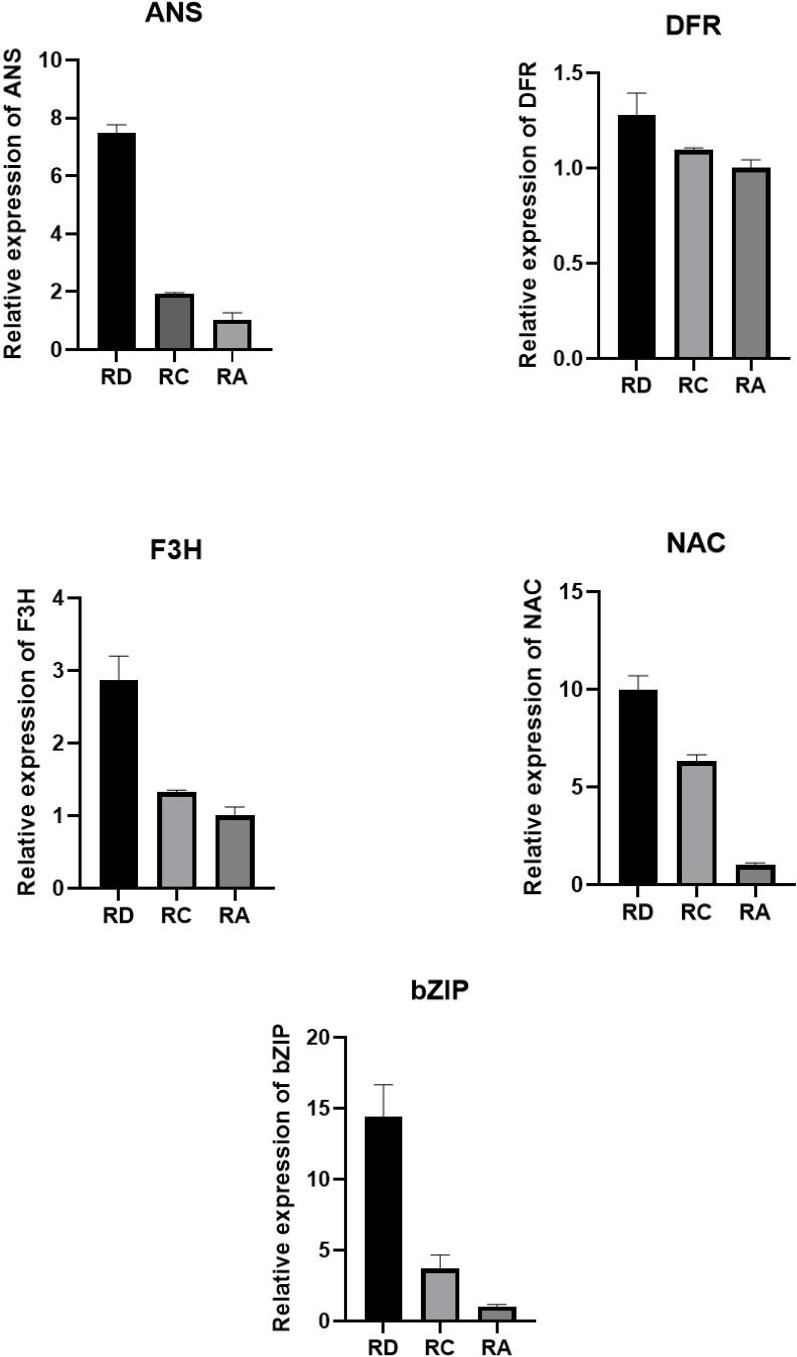
qRT-PCR results.

To validate the expression patterns of key genes identified from the co-expression network analysis, including enzyme-encoding genes and potential transcription factors, we performed quantitative reverse transcription PCR (qRT-PCR) analysis on three rose cultivars (RD, RC, and RA). Total RNA was extracted from petal samples using the SteadyPure Plant RNA Extraction Kit (Accurate Biotechnology, Hunan, China). The purified RNA was then reverse-transcribed into first-strand cDNA using the Evo M-MLV Reverse Transcription Premix Kit (with gDNA Remover for qPCR) Ver.2 (Accurate Biotechnology, Hunan, China). The qRT-PCR reaction mixture contained 5 μL of 2× SYBR Green Pro Taq HS Premix (Accurate Biotechnology, Hunan, China), 1 μL of cDNA template, 0.5 μL each of forward and reverse primers, and 3 μL of RNase-free water, in a final volume of 10 μL. Reactions were performed on a Fluorescent Quantitative PCR Detection System (Bioer Technology, Hangzhou, China) under the following program: initial denaturation at 95 °C for 30 s, followed by 40 cycles of 95 °C for 5 s and 60 °C for 30 s. At least four biological replicates were included for each sample. The rose GAPDH gene was used as an internal reference for normalization. Gene-specific primers used for qRT-PCR are listed in **Supplementary Table S3**. The relative expression levels of target genes were calculated using the 2^−ΔΔCt^ method (**Figure 1**).

## 3 Results

### 3.1 Metabolic analysis

**Figure 2:**
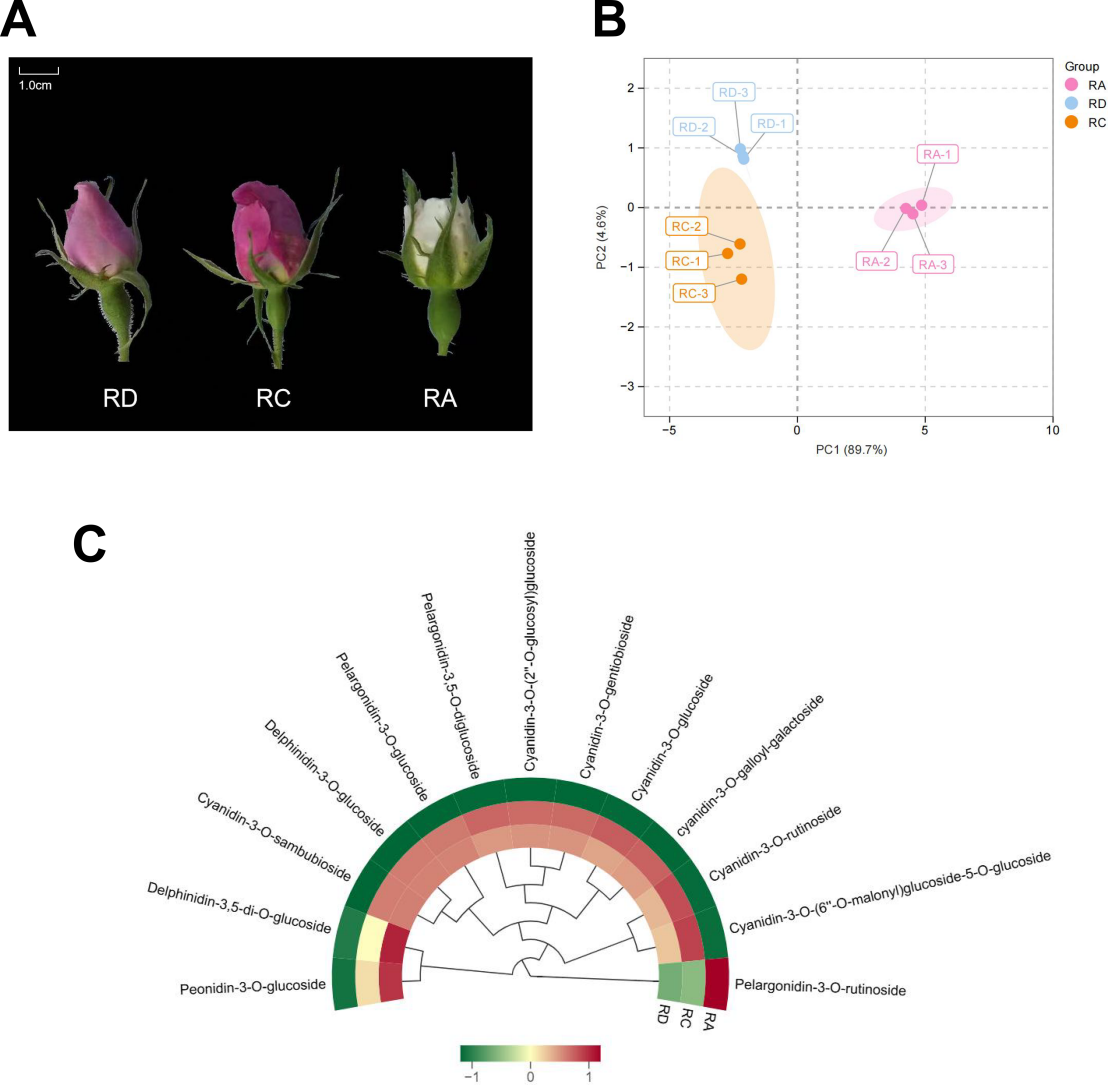
Overview of metabolomics results. **(A)** Three heritage rose samples. **(B)** Principal Component Analysis (PCA) of Anthocyanin Samples. **(C)** Cluster analysis heatmap of 13 anthocyanin contents.

To systematically profile anthocyanin accumulation patterns across rose varieties, we analyzed three cultivars—RD(pink), RC (purplish-red), and RA (white) (**Figure 2A**)—using UPLC-MS/MS. As illustrated in **Figures 2B**, the samples exhibited clear separation in multivariate space: RD and RC clustered closely, indicating similar metabolite profiles, while RA was distinctly separated from both.

A total of 13 anthocyanins were identified (**Figure 2C**, **Supplementary Table S1**), including Cyanidin-3-O-(2’’-O-glucosyl)glucoside, Cyanidin-3-O-(6’’-O-malonyl)glucoside-5-O-glucoside, Cyanidin-3-O-gentiobioside, Cyanidin-3-O-glucoside, Cyanidin-3-O-rutinoside, Cyanidin-3-O-sambubioside, Delphinidin-3,5-di-O-glucoside, Delphinidin-3-O-glucoside, Pelargonidin-3,5-O-diglucoside, Pelargonidin-3-O-glucoside, Pelargonidin-3-O-rutinoside, Peonidin-3-O-glucoside, and cyanidin-3-O-galloyl-galactoside. Most of the detected anthocyanins were derived from cyanidin.

In terms of abundance, RC showed the highest total anthocyanin content, exceeding that of RD by 6.9% and RA by approximately fivefold. RD accumulated significantly higher levels of Peonidin-3-O-glucoside and Delphinidin-3,5-di-O-glucoside compared to RC and RA, whereas RA was notably rich in Pelargonidin-3-O-rutinoside.

These anthocyanins are typical of rose petals and predominantly undergo glycosylation (e.g., glucosylation, rutinosylation) or acylation (e.g., malonylation). Glycosylation enhances water solubility and stability, facilitating vacuolar storage and influencing color expression and bioactivity. Acylation further improves stability, reducing degradation during processing and storage. Together, these natural modifications strengthen the potential of rose anthocyanins as functional food ingredients.

### 3.2 Proteomic analysis

**Figure 3:**
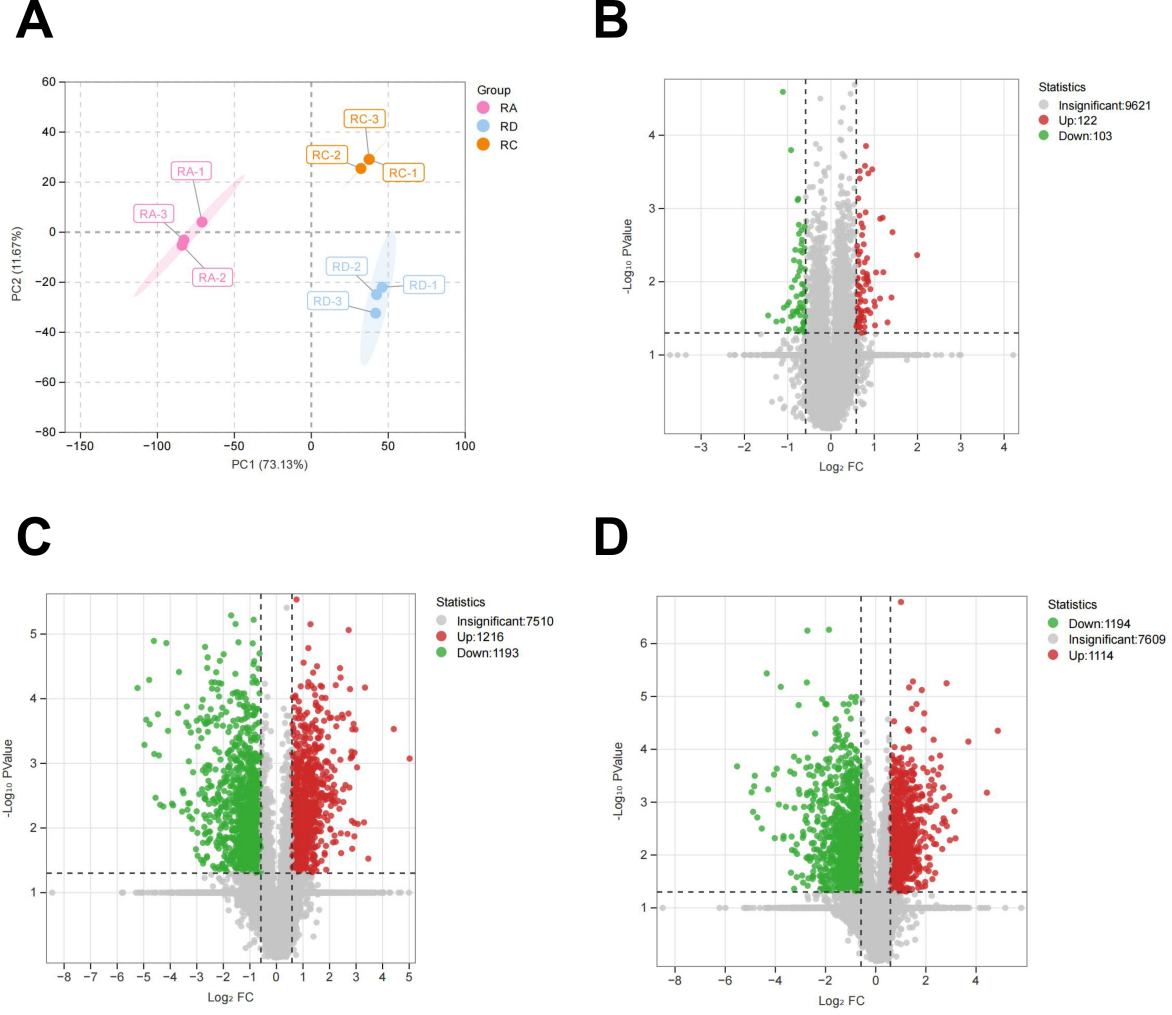
Overview of proteomics results. **(A)** PCA score plot. **(B)** Volcano plots in RC vs RD. **(C)** Volcano plots in RA vs RD. **(D)** Volcano plots in RA vs RC. .

To elucidate the molecular mechanisms underlying anthocyanin biosynthesis in roses, we conducted a comprehensive proteomic analysis of the three cultivars. Across the nine samples analyzed, a total of 77,499 peptides and 9,924 proteins were identified. Principal component analysis (PCA) revealed clear sample clustering, with RD and RC exhibiting high similarity in protein composition, while RA was distinctly separated from both (**Figure 3A**).

To further explore varietal regulatory differences, we established three comparative groups: RC vs. RD, RA vs. RD, and RA vs. RC. Using a threshold of p < 0.05 and |Log_2_FC| ≥ 1.5, we identified 225, 2409, and 2308 differentially expressed proteins (DEPs) in the three comparisons, respectively. Specifically, RC vs. RD showed 122 up-regulated and 103 down-regulated DEPs; RA vs. RD contained 1215 up-regulated and 1193 down-regulated DEPs; and RA vs. RC included 1114 up-regulated and 1194 down-regulated DEPs (**Figures 3B–D**).

**Figure 4:**
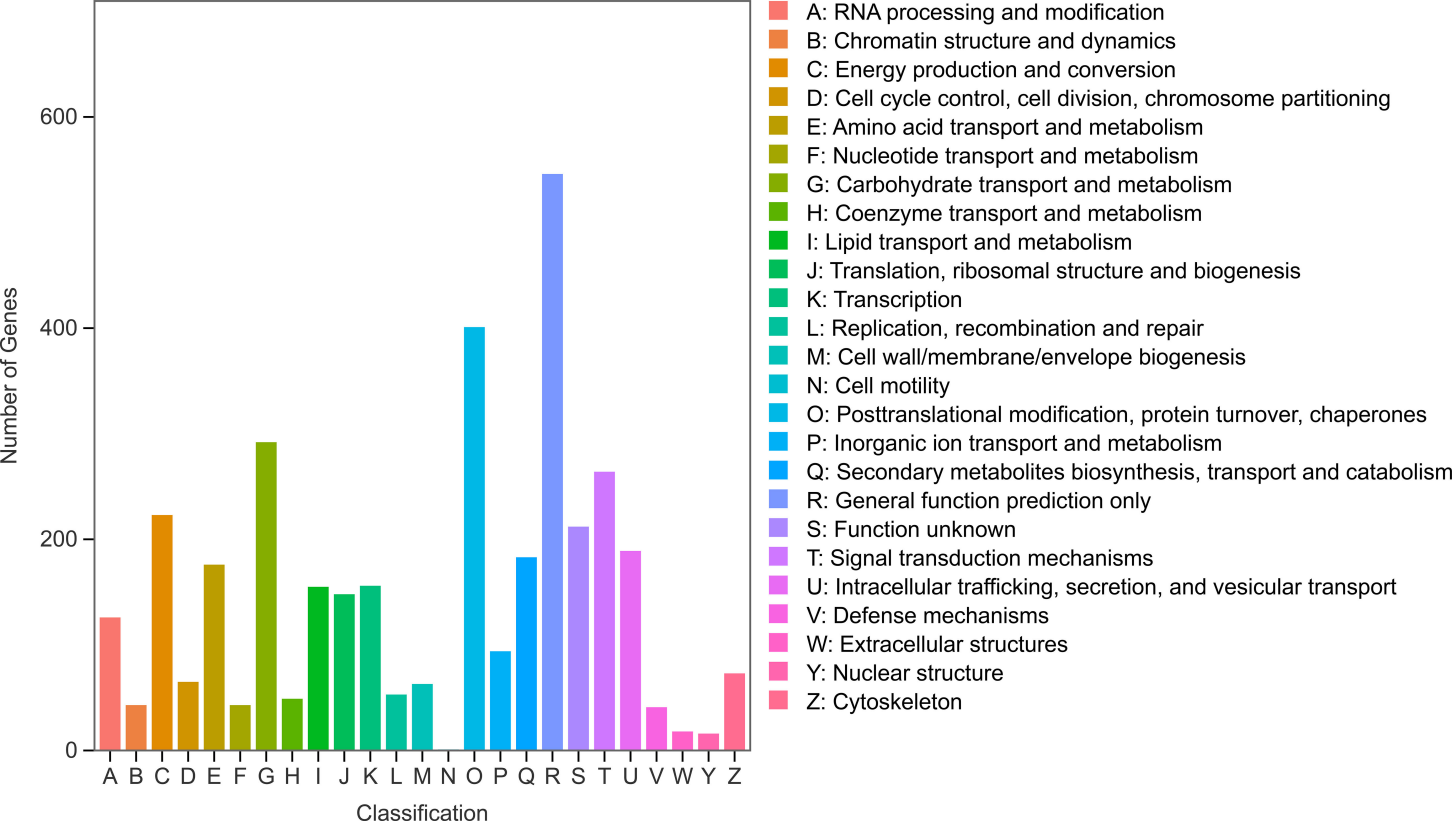
KOG analysis of proteins.

Functional annotation of DEPs was performed using the Eukaryotic Orthologous Groups (KOG) database (**Figure 4**). KOG analysis highlighted that category Q (secondary metabolite biosynthesis, transport, and catabolism) encompasses the full regulatory spectrum of plant secondary metabolites—including anthocyanins, which are typical representatives of such compounds. Secondary metabolites play key roles in plant adaptation to external stimuli (e.g., UV protection, pollinator attraction) and internal physiological regulation. The biosynthesis, intracellular transport, and degradation of anthocyanins are all governed by functional modules within the Q category. These findings suggest that expression divergence in Q-related functional modules may underlie the proteomic variation and differential anthocyanin accumulation observed among rose varieties.

### 3.3 Conjoint analysis

**Figure 5:**
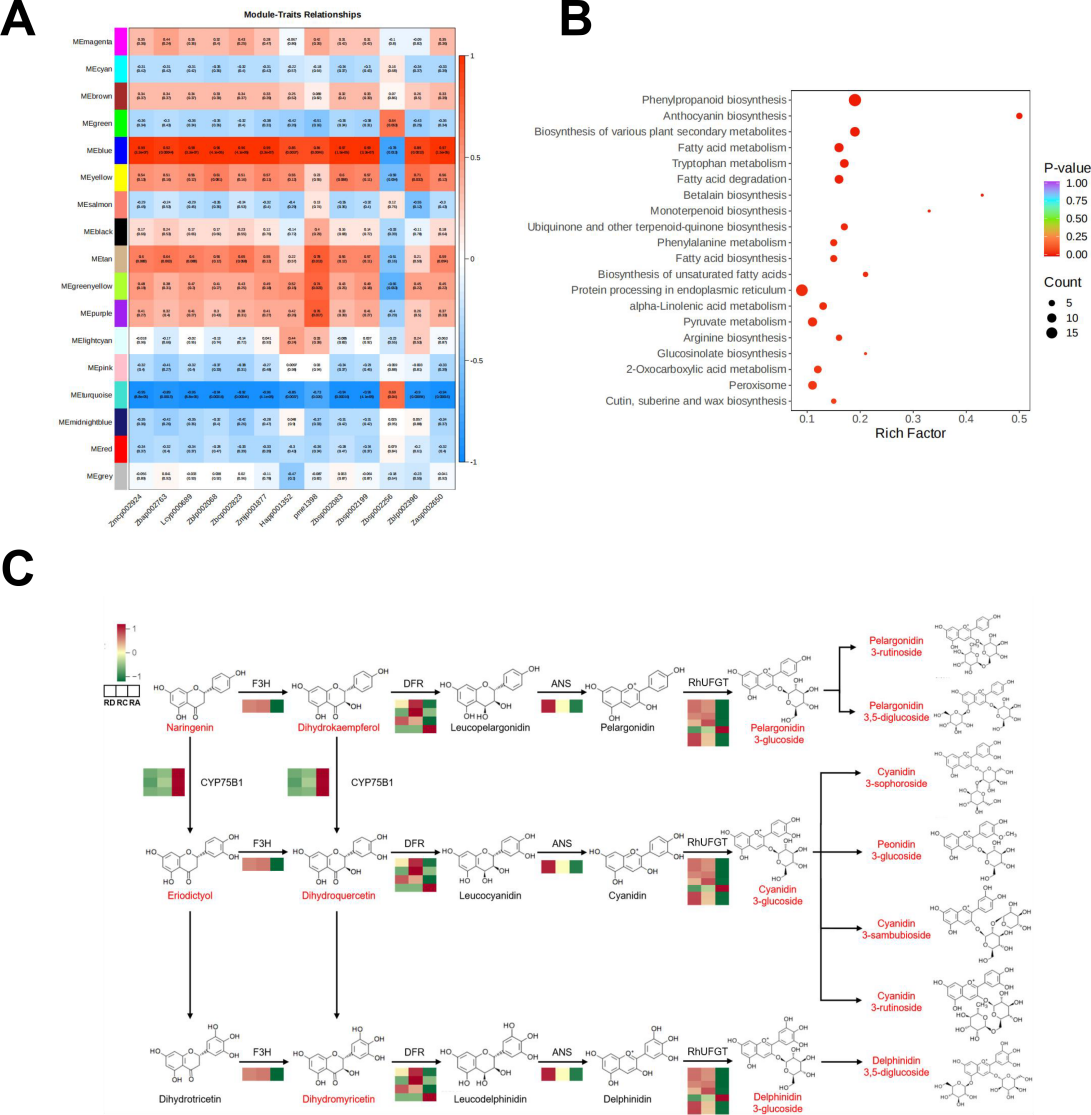
Overview of the results of the combined analysis of metabolomics and proteomics. **(A)** The heat map showing the correlation between each expression module and anthocyanins. **(B)** KEGG enrichment analysis of the proteins in MEbiue. **(C)** anthocyanin biosynthesis. Differential compounds were marked in red, the scale represented the relative expression level of proteins as increasing from green to red. Each scale had three grids presented as “RD” “RC” and “RA” respectively, from left to right.

**Figure 6:**
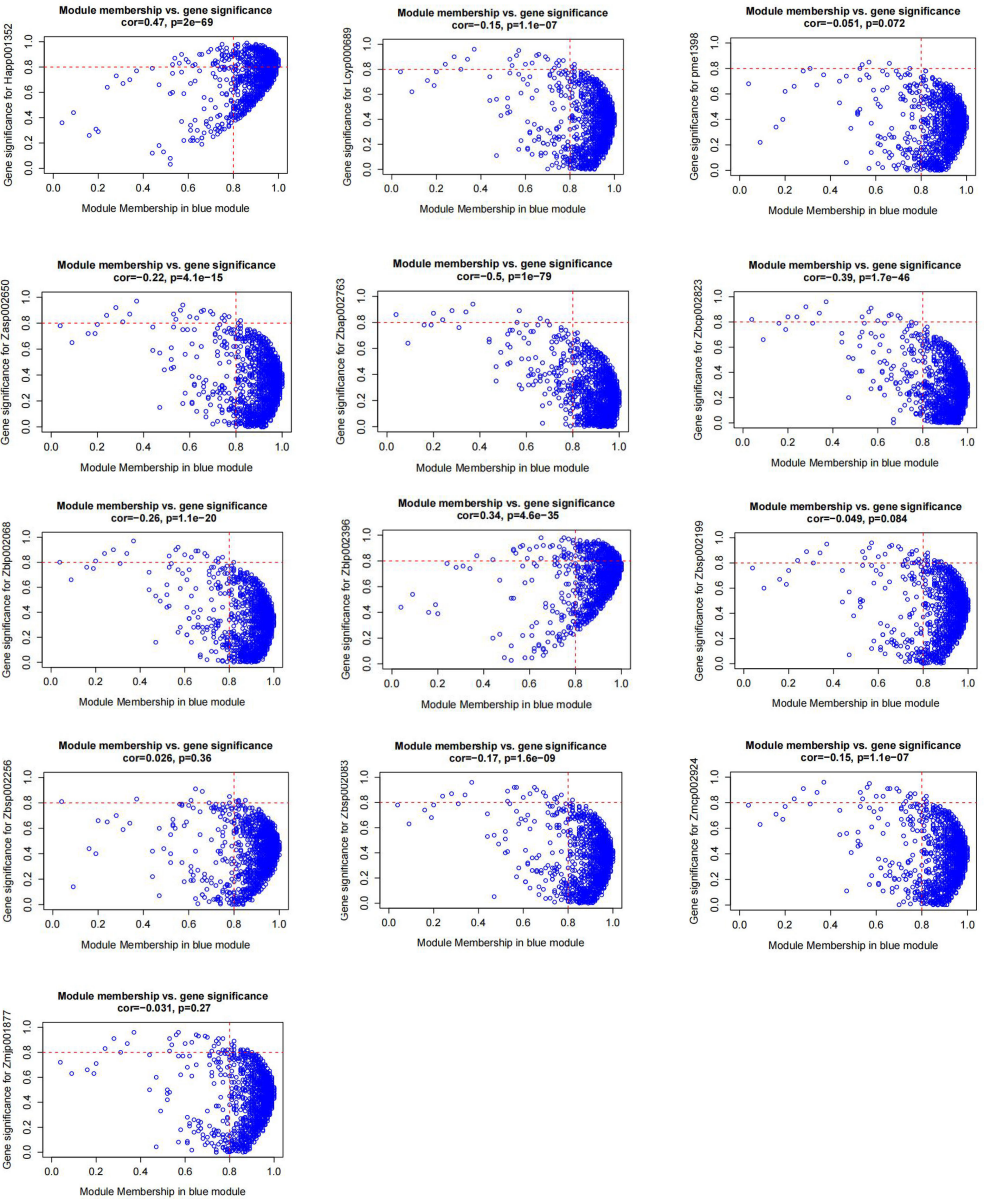
MM-GS scatter plot of MEblue.

To investigate the relationship between metabolite profiles and protein expression, we integrated anthocyanin content and proteomic data through Weighted Gene Co-expression Network Analysis (WGCNA) (**Figure 5A** and **Figure 6**). This approach identified 17 co-expression modules, among which the MEblue module exhibited the strongest overall correlation with anthocyanin accumulation. Specifically, this module showed Pearson correlation coefficients of 0.99, 0.92, 0.99, 0.96, 0.96, 0.99, 0.85, 0.84, 0.97, 0.99, -0.78, 0.89, and 0.97 with individual anthocyanin compounds, respectively.

Within the MEblue module, genes associated with multiple cyanidin, delphinidin, and pelargonidin derivatives—including Cyanidin-3-O-(2’’-O-glucosyl) glucoside, Delphinidin-3,5-di-O-glucoside, and Pelargonidin-3-O-glucoside—were highly expressed in RD and RC samples but showed markedly lower expression in RA. In contrast, genes linked specifically to Pelargonidin-3-O-rutinoside were more abundant in RA and relatively suppressed in RD and RC, suggesting a distinct regulatory pattern for this compound.

KEGG pathway enrichment analysis revealed that proteins in the MEblue module were significantly associated with phenylpropanoid biosynthesis, anthocyanin biosynthesis, and the biosynthesis of various plant secondary metabolites (**Figure 5B**). Based on these findings, we reconstructed the anthocyanin biosynthetic pathway, mapped the key enzymes, and visualized their expression patterns through heatmaps, providing a comprehensive view of the regulatory network underlying anthocyanin variation among rose varieties (**Figure 5C**).

### 3.4 Machine learning

**Figure 7:**
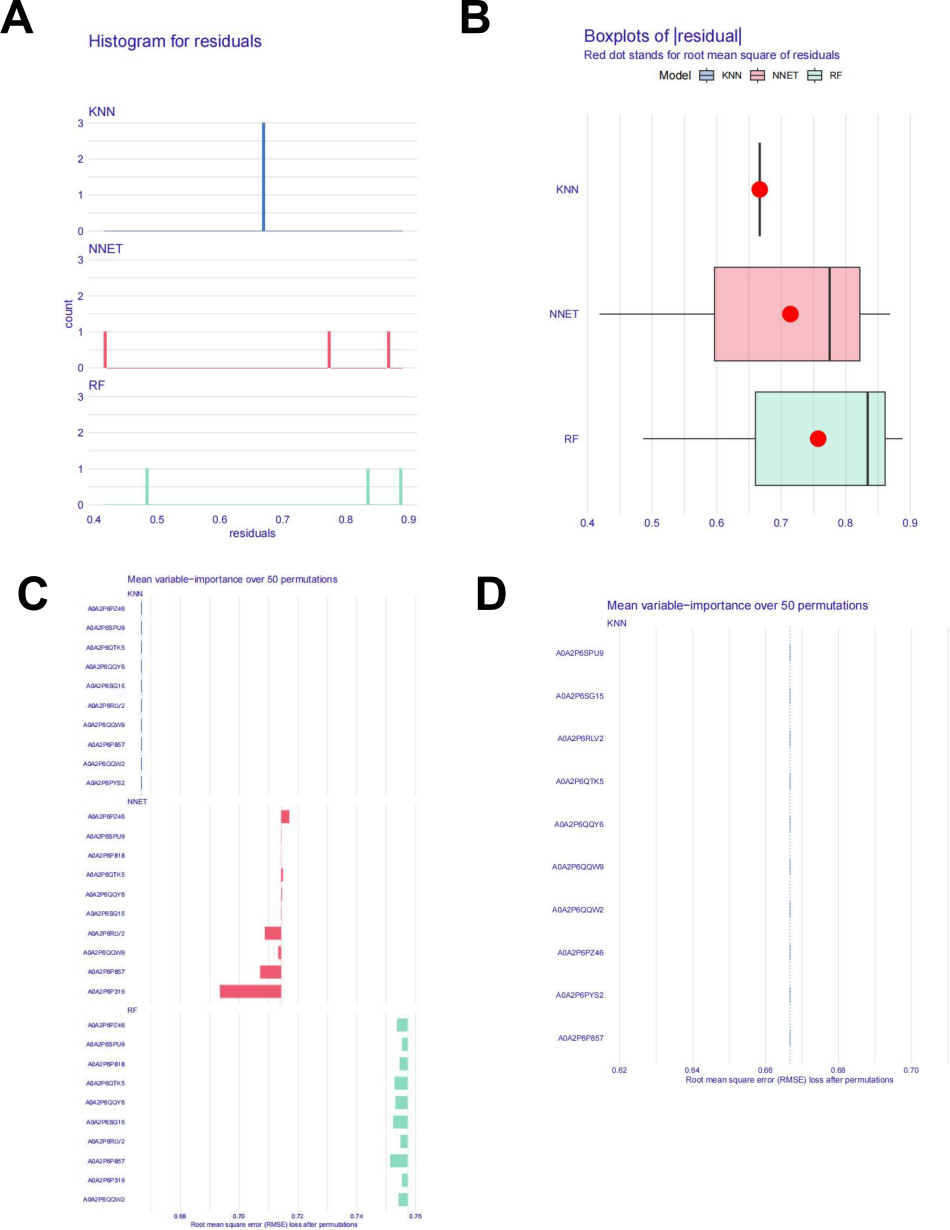
Overview of Machine Learning Results **(A)** histogram for residuals. **(B)** Boxplots of absolute residuals. **(C)** The top ten key proteins of the three models. **(D)** The top ten key proteins in the KNN model.

We selected 16 proteins associated with key enzyme genes in anthocyanin biosynthesis as candidate proteins, with the set of differentially expressed proteins serving as the background library.Three machine learning models—Random Forest (RF), K-Nearest Neighbors (KNN), and Neural Network (NNET)—were constructed and evaluated. Model performance was evaluated via 5-fold cross-validation repeated 10 times, and the mean squared error (MSE) and R² were calculated. As shown in **Figures 7A, B**, the KNN model exhibited smaller residuals, lower deviation, and greater stability compared to the other models. The K-Nearest Neighbors (KNN) model (with k=3) was trained using the expression profiles of differentially expressed proteins across the three biological replicates of each variety.Based on these performance metrics, we ultimately identified the ten proteins selected by the KNN model as key candidates for subsequent functional analysis (**Figures 7C, D**, **Supplementary Table S2**).

### 3.5 Transcription factor regulation

**Figure 8:**
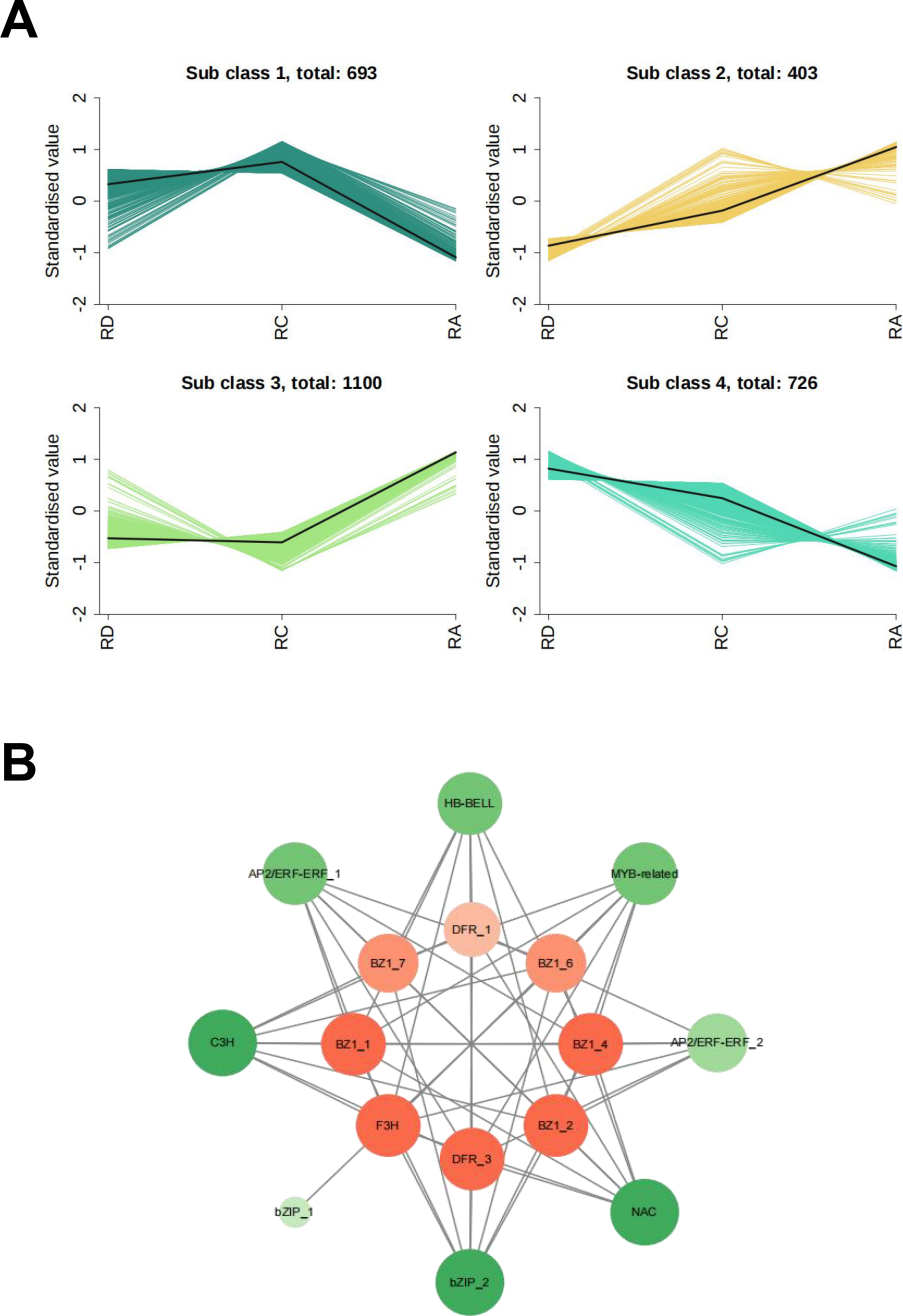
Overview of transcription factor correlation analysis. **(A)** Four distinct expression patterns of proteins revealed by K-means clustering. **(B)** Co-expression correlation networks and the connection among transcription factors (TFs) and structural proteins involved in anthocyanin biosynthesis.

Through integrated protein clustering (K-means) and metabolome–proteome correlation analysis, we identified transcription factors (TFs) associated with the anthocyanin biosynthesis pathway to elucidate upstream transcriptional regulation. Four distinct protein subclusters were resolved: subcluster 1 contained 693 proteins, subcluster 2 contained 403, subcluster 3 contained 1100, and subcluster 4 contained 726, each exhibiting unique expression trends across varieties (**Figure 8A**).

Notably, proteins in subcluster 1 showed significantly higher expression in RD and RC compared to RA. Their expression pattern closely mirrored the total anthocyanin content across the three varieties and aligned with the expression trends of key proteins from the MEblue module. This consistency suggests that subcluster 1 proteins are likely involved in anthocyanin synthesis and accumulation, potentially including regulatory TFs.

By integrating subcluster 1 with the MEblue module, we screened out proteins, and predicted transcription factors based on the sequences of these proteins. Ultimately, eight transcription factors were identified from different families, including members of the *C3H*, *bZIP*, *HB-BELL*, *AP2/ERF-ERF*, *NAC*, and *MYB-related* families. A co-expression network linking these TFs with the 10 previously identified key proteins was constructed and visualized in Cytoscape (**Figure 8B**). Among them, *C3H*, *bZIP_2*, and *NAC* displayed high network connectivity, indicating their potential as central regulators of anthocyanin-related protein expression.

## 4 Discussion

### 4.1 Association between anthocyanin composition and flower color/function

The composition of anthocyanins in different rose varieties is closely associated with their floral coloration. Metabolomic analysis revealed that RC, with its high total anthocyanin content—primarily composed of cyanidin and pelargonidin derivatives—displays a deep purplish-red hue. In contrast, RD exhibits a pink coloration, which correlates with its predominant accumulation of delphinidin- and peonidin-based anthocyanins (including Delphinidin-3,5-di-O-glucoside, Delphinidin-3-O-glucoside, and Peonidin-3-O-glucoside), coupled with relatively low levels of pelargonidin and cyanidin types. RA, appearing white, showed marked accumulation only of Pelargonidin-3-O-rutinoside, while other anthocyanins were minimally detected.

The identified anthocyanins predominantly exist in glycosylated or acylated forms, which is crucial for their functionality. Glycosylation, such as the attachment of glucose or rutinose, is not merely a terminal decoration but a critical determinant of molecular stability, water solubility, and proper vacuolar sequestration—factors essential for color manifestation and cellular homeostasis. Acylation, particularly malonylation, adds another layer of stability, protecting the anthocyanin skeleton from degradation during post-harvest processing and storage. From an applied perspective, these natural modifications are highly advantageous. They enhance the shelf-life and color fidelity of rose-based pigments, thereby increasing their industrial viability as natural colorants and bioactive ingredients in functional foods.

Studies have confirmed that among various anthocyanin types, delphinidin exhibits the most potent *in vitro* antioxidant and anti-inflammatory activities. Based on the metabolomic findings of this study, if the objective in developing functional beverages or related products is to maximize delphinidin content for enhanced antioxidant and anti-inflammatory efficacy, it is recommended to select rose varieties such as RD as the raw material. Although the total anthocyanin content in RD is lower than that in RC, its abundance of delphinidin-type anthocyanins (e.g., Delphinidin-3,5-di-O-glucoside and Delphinidin-3-O-glucoside) is significantly higher than in the other two varieties, thereby offering a robust source of delphinidin for product formulation.

If the goal of functional product development is to leverage the synergistic effects of multiple anthocyanins for broader physiological benefits, it is recommended to select deeply pigmented purplish-red rose varieties such as RC as raw materials. RC not only exhibits the highest total anthocyanin content but also contains a diverse profile of anthocyanin types, including cyanidin, pelargonidin, delphinidin, and peonidin derivatives. These compounds are known to act synergistically in scavenging free radicals, enhancing immune response, and protecting visual health, thereby offering comprehensive functional benefits that align with the multi-target health demands of modern consumers.

In contrast, RA holds relatively limited value for anthocyanin-centered functional products due to its low total anthocyanin content and restricted compositional diversity. It may be better suited for applications with low anthocyanin requirements—such as pastry decoration or flavor enhancement—or for non-food uses including cosmetic formulations and ornamental purposes. Additionally, RA could serve as a material base for metabolic engineering aimed at enhancing anthocyanin accumulation without introducing undesirable coloration.

### 4.2 Functional analysis of key proteins in anthocyanin synthesis

The anthocyanin biosynthesis pathway diverges into three major branches responsible for the production of pelargonidin, cyanidin (which can be methylated to peonidin), and delphinidin (methylated to form petunidin and malvidin), respectively (Jaakola, 2013). This process is coordinated by key structural genes, including *DFR*, *ANS*, *F3H*, *CYP75B1*, and *RhUFGT* (Goswami et al., 2018; Iaria et al., 2016; Jaakola, 2013; Koes et al., 2005).

Specifically, naringenin is converted to eriodictyol by CYP75B1, and further transformed into dihydrotricetin. F3H then catalyzes the formation of dihydrokaempferol, dihydroquercetin, and dihydromyricetin from naringenin, eriodictyol, and dihydrotricetin. DFR further reduces these intermediates to corresponding colorless anthocyanins (such as Leucopelargonidin, Leucocyanidin, and Leucodelphinidin); ANS catalyzes its oxidation to produce colored precursors such as pelargonidin, cyanidin, and delphinidin; Finally, stable anthocyanins are formed under the action of glycosyltransferases such as RhUFGT, including Pelargonidin-3-glucoside, cyanidin-3-glucoside, and delphinidin-3-glucoside, which are then modified to produce a series of anthocyanins.

Through machine learning-based screening, we identified ten key proteins involved in anthocyanin biosynthesis: RhUFGT_1, RhUFGT_2, RhUFGT_4, RhUFGT_5, RhUFGT_6, RhUFGT_7, F3H, DFR_1, DFR_3, and CYP75B1_3. Among them, RhUFGT_1, RhUFGT_2, RhUFGT_4, F3H, and DFR_3 showed similarly high expression levels in both RD and RC. RhUFGT_6 and RhUFGT_7 were also expressed in both varieties but were significantly higher in RD than in RC. In contrast, DFR_1 was notably more abundant in RC than in RD and RA, while CYP75B1_3 was markedly upregulated in RA compared to the other two varieties.

The expression profiles of these key proteins are consistent with the observed anthocyanin accumulation pattern, wherein RD and RC exhibited substantially higher anthocyanin content than RA. We thus propose that RhUFGT_1, RhUFGT_2, RhUFGT_4, F3H, and DFR_3 likely function as positive regulators of anthocyanin synthesis. In contrast, the higher abundance of CYP75B1_3 in the low-anthocyanin variety RA presents an intriguing finding. CYP75B1 typically promotes the synthesis of delphinidin precursors. Its negative correlation with total anthocyanin content here may suggest a feedback inhibition mechanism, a diversion of flux into competing branch pathways (e.g., towards other flavonoids), or potential functional diversification of specific isoforms in roses. Further investigation into the specific catalytic activity and subcellular localization of this CYP75B1_3 isoform is needed to clarify its role.

### 4.3 Expansion of the transcription factor regulatory network

While previous studies have established the central role of *R2R3-MYB* transcription factors such as *RcMYB1* in regulating anthocyanin biosynthesis in roses (He et al., 2023), the involvement of TF families beyond *MYB* has remained less clear. In this study, K-means clustering analysis also identified *MYB* family TFs (including *R2R3-MYB* types) in subcluster 1, and their expression patterns aligned with total anthocyanin accumulation. Correlation analysis between the key anthocyanin-related proteins and identified TFs revealed significant positive associations with several key enzymes, including F3H, DFR_3, and RhUFGT_2. These findings are consistent with earlier reports and not only validate the reliability of the TF identification approach used here but also support functional inference for other previously uncharacterized TFs.

Co-expression network analysis further suggested that *NAC*, *bZIP_2*, and *C3H* transcription factors may also participate in the regulation of anthocyanin synthesis in roses. Previous studies have demonstrated that in strawberry, the *bZIP* transcription factor *FaTRAB1* responds to ABA signaling and binds to the promoters of anthocyanin structural genes in a tissue-specific manner. Concurrently, it interacts synergistically with transcription factors such as *NAC* to activate the expression of *FaMYB10* and downstream structural genes, thereby directionally promoting anthocyanin biosynthesis in leaves (Yang et al., 2025). Furthermore, in apple, ABA can induce the formation of a protein complex between the *NAC*-family transcription factor *MdNAC1* and the *bZIP* factor *MdbZIP23*. This complex subsequently binds to the promoters of *MdMYB10* and *MdUFGT*, activating their transcription and reinforcing the MBW regulatory pathway, which enhances anthocyanin accumulation in red-fleshed apples (Liu et al., 2023). These examples provide a plausible framework for how NAC and bZIP TFs might integrate into the anthocyanin regulatory network in roses. They could act upstream of or in parallel with the core MBW complex, possibly mediating environmental or hormonal signals (like ABA) to fine-tune anthocyanin biosynthesis. Based on these findings, we hypothesize that *NAC* and *bZIP* transcription factors may similarly play important regulatory roles in anthocyanin biosynthesis in roses, though their specific mechanisms require further investigation. Additionally, research has indicated that *C3H*-type transcription factors function as key regulators in anthocyanin synthesis in Lycoris radiata (Wang et al., 2021). In roses, this study preliminarily proposes that *C3H*-type transcription factors may also be involved in the regulatory network of anthocyanin biosynthesis, and their precise mechanisms of action likewise warrant further elucidation. Based on these predictions, future work will include experimental validation to elucidate the specific roles of these candidate TFs in the anthocyanin regulatory network.

In summary, this study systematically investigated the variation in anthocyanin composition and abundance among three rose varieties—RD, RC, and RA—using integrated metabolomic and proteomic approaches. We identified multiple key enzymes and candidate transcription factors potentially involved in anthocyanin biosynthesis, establishing a molecular framework for future functional studies and the genetic improvement of rose quality. Functional validation of the predicted transcription factors will be carried out in subsequent research.

## 5 Conclusion

This study systematically compared the composition and biosynthesis of anthocyanins in the petals of *Rosa alba*, *Rosa damascena*, and *Rosa centifolia* through integrated metabolomic and proteomic analyses. The results reveal a strong association between anthocyanin composition and floral coloration: RC, with its high accumulation of cyanidin and pelargonidin derivatives, displays a purplish-red hue; RD, rich in delphinidin and peonidin types, shows a pink coloration; whereas RA, containing only elevated levels of Pelargonidin-3-O-rutinoside, appears white. These distinct metabolic profiles provide a basis for selecting rose varieties for specific applications, ranging from natural colorants to potential functional ingredients.

At the molecular level, integrated WGCNA and machine learning approaches identified ten key proteins—including RhUFGT, F3H, DFR, and CYP75B1—in anthocyanin biosynthetic pathways. Their expression patterns across the three rose varieties aligned closely with respective anthocyanin accumulation profiles. Furthermore, this study expands the current understanding of anthocyanin transcriptional regulation by revealing that beyond the established MYB factors, additional transcription factors—including *NAC*, *bZIP_2*, and *C3H*—may participate in regulating anthocyanin synthesis. We propose that these TFs could interact with or modulate the core MBW complex, integrating internal or external signals to fine-tune pigment production. These findings provide strategic direction for subsequent functional validation studies.

In summary, this study demonstrates the effectiveness of integrated metabolomics and proteomics in elucidating the complex regulatory network of anthocyanin biosynthesis in edible rose petals. We identified *Rosa centifolia* as the most promising variety for anthocyanin extraction and revealed key structural proteins and transcription factors governing accumulation. These findings deepen our understanding of the biochemical and genetic mechanisms underlying anthocyanin production in roses and provide a molecular foundation for breeding strategies aimed at enhancing their value as sources of natural pigments and functional food ingredients. Future work will focus on the functional characterization of key transcription factors and the application of these findings in the development of rose-based functional products.

## Data Availability

The datasets presented in this study can be found in online repositories. The names of the repository/repositories and accession number(s) can be found below: https://www.iprox.cn/page/project.html?id=IPX0011557000.
