# Supplementary figures and images for: Machine learning integrates metabolomics and proteomics to identify key regulators of anthocyanin biosynthesis in edible rose petals
Source: Front Plant Sci. 2026 Mar 13;17:1751780. doi: 10.3389/fpls.2026.1751780 (PMC13021854; doi:10.3389/fpls.2026.1751780)

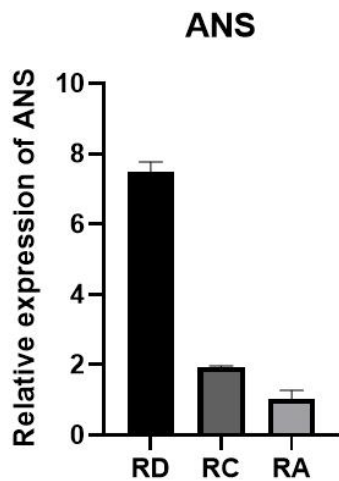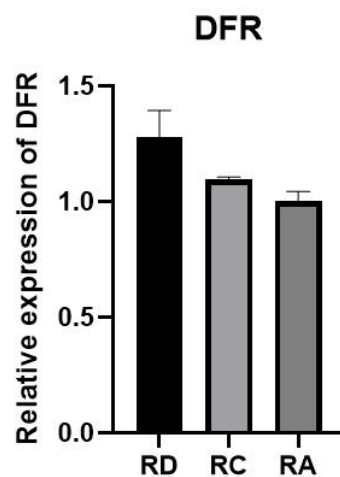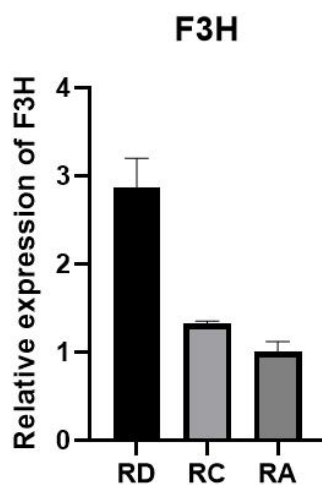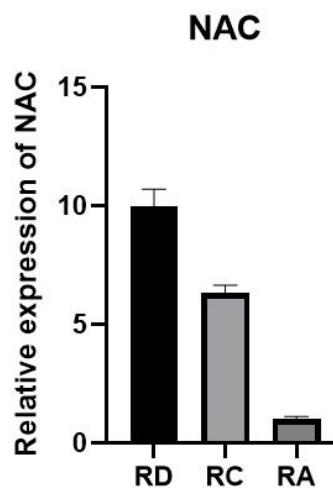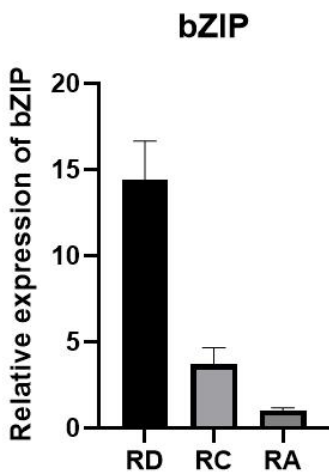

Supplement: Supplementary file 1 [file Image1.pdf]

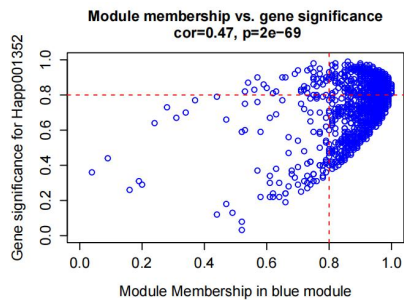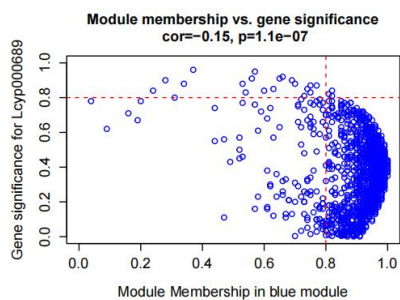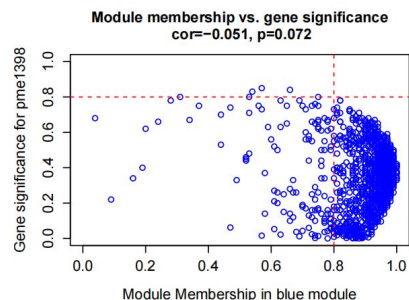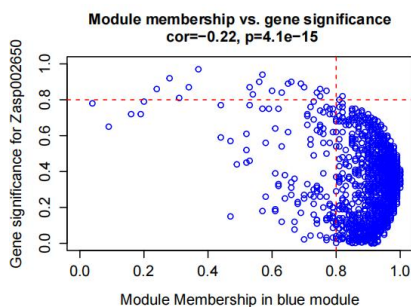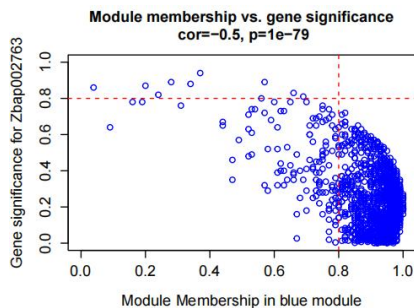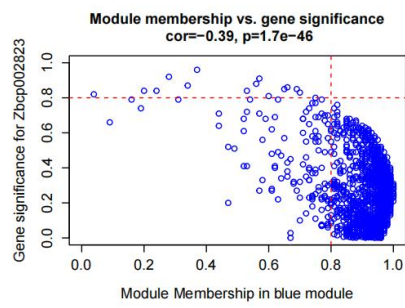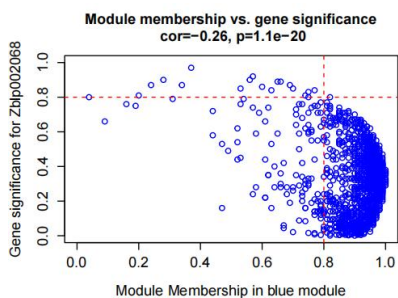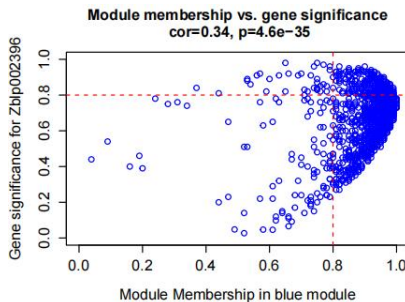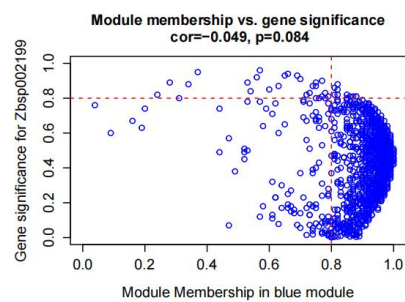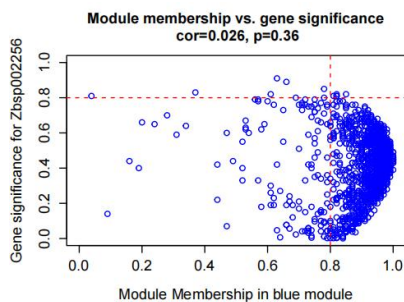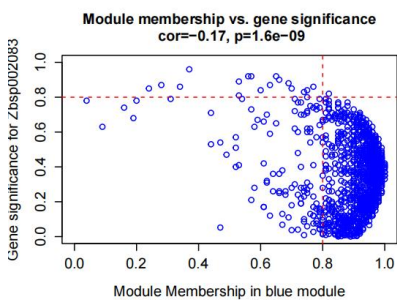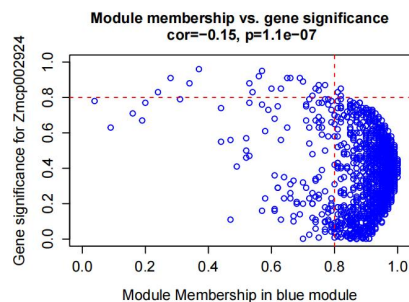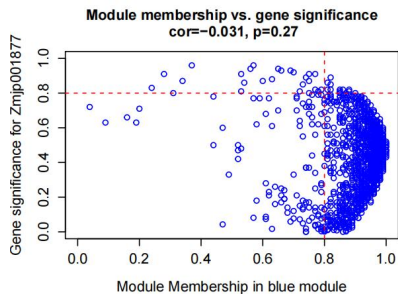

Supplement: Supplementary file 2 [file Image2.pdf]
