# Supplementary material for: Machine learning integrates metabolomics and proteomics to identify key regulators of anthocyanin biosynthesis in edible rose petals
Source: Front Plant Sci. 2026 Mar 13;17:1751780. doi: 10.3389/fpls.2026.1751780 (PMC13021854; doi:10.3389/fpls.2026.1751780)
Supplement: Supplementary file 3 [file Table1.docx]

| ID | Compounds | Relative expression | | |
| --- | --- | --- | --- | --- |
|  |  | RD | RC | RA |
| Zmcp002924 | Cyanidin-3-O-(2''-O-glucosyl)glucoside | 74171239.36 | 81864558.06 | 4744334.381 |
| Zbap002763 | Cyanidin-3-O-(6''-O-malonyl)glucoside-5-O-glucoside | 802073.5332 | 1119403.834 | 58070.40567 |
| Lcyp000689 | Cyanidin-3-O-gentiobioside | 84097349.6 | 93132745.54 | 5186702.731 |
| Zblp002068 | Cyanidin-3-O-glucoside | 7032769.362 | 8404913.086 | 573782.0235 |
| Zbcp002823 | Cyanidin-3-O-rutinoside | 6128548.811 | 7917535.319 | 627096.0048 |
| Zmjp001877 | Cyanidin-3-O-sambubioside | 9501151.636 | 9536260.621 | 361275.3619 |
| Zasp002650 | cyanidin-3-O-galloyl-galactoside | 11735993.27 | 13713830.25 | 70153.26213 |
| Happ001352 | Delphinidin-3,5-di-O-glucoside | 3277555.051 | 1709261.655 | 105168.0293 |
| pme1398 | Delphinidin-3-O-glucoside | 30097620.66 | 30180564.87 | 23903766.42 |
| Zbsp002083 | Pelargonidin-3,5-O-diglucoside | 4366651.009 | 4963899.925 | 32623.23013 |
| Zbsp002199 | Pelargonidin-3-O-glucoside | 32537864.57 | 33289928.35 | 638490.9207 |
| Zbsp002256 | Pelargonidin-3-O-rutinoside | 12103360.65 | 12466407.93 | 18161215.03 |
| Zblp002396 | Peonidin-3-O-glucoside | 13740202.18 | 11449236.71 | 7777542.444 |

Table S1. The expression levels of 13 anthocyanins.

| ID | Accession | Relative expression | | |
| --- | --- | --- | --- | --- |
|  |  | RD | RC | RA |
| A0A2P6PZ46 | BZ1_1 | 3.26360 | 3.11246 | 1.32649 |
| A0A2P6QQW2 | BZ1_2 | 5.71208 | 5.29609 | 0.94406 |
| A0A2P6QQW9 | BZ1_4 | 1.41814 | 1.67994 | 0.59018 |
| A0A2P6QQY6 | BZ1_5 | 1.09989 | 1.32732 | 2.06134 |
| A0A2P6QTK5 | BZ1_6 | 1.27375 | 1.17655 | 0.93856 |
| A0A2P6SPU9 | BZ1_7 | 0.22026 | 0.15322 | 0.00000 |
| A0A2P6RLV2 | F3H | 9.70024 | 9.81209 | 6.24124 |
| A0A2P6PYS2 | FDFR_1 | 9.09409 | 11.94666 | 5.44981 |
| A0A2P6SG15 | FDFR_3 | 1.50259 | 1.21925 | 0.00000 |
| A0A2P6P857 | CYP75B1_3 | 0.71155 | 0.77034 | 2.55862 |

Table S2. The ten key proteins selected by machine learning

| **Genes name** | **Forward sequence 5’→3’** | **Reverse sequence 5’→3’** |
| --- | --- | --- |
| GAPDH | TGAAGGGTGGTGCCAAGAA | AAGGGGAGCAAGACAGTTGG |
| ANS | AGCTCATGGAACGGGTCAAG | TTGCCCGGAAGCATTGTTTG |
| DFR | CGTAGGTTCATGGCTCGTCA | TCCACAGCGTCAAGTGAGTC |
| F3H | TTCAAGAACGCCGATCACCA | TGGCTCCTCCAGAATAGGCT |
| NAC | AACGCGAACGAGTGGTACTT | GCGTCTTCCTCATCCCTACG |
| bZIP | GAACAGGAGCGCTTCAGAT | AGCTTGGTCTTGAGGAAGGC |

Table S3. Primers for real-time quantitative PCR.
